# Identification of gene profiles related to the development of oral cancer using a deep learning technique

**DOI:** 10.1186/s12920-023-01462-6

**Published:** 2023-02-27

**Authors:** Leili Tapak, Mohammad Kazem Ghasemi, Saeid Afshar, Hossein Mahjub, Alireza Soltanian, Hassan Khotanlou

**Affiliations:** 1 Department of Biostatistics, School of Public Health and Modeling of Noncommunicable Diseases Research Center, Hamadan University of Medical Sciences, Hamadan, Iran; 2 Department of Biostatistics, School of Public Health, Hamadan University of Medical Sciences, Hamadan, Iran; 3 Research Center for Molecular Medicine, Hamadan University of Medical Sciences, Hamadan, Iran; 4 Department of Computer Engineering, Bu-Ali Sina University, Hamadan, Iran

**Keywords:** Oral cancer, Deep learning, Gene expression

## Abstract

**Background:**

Oral cancer (OC) is a debilitating disease that can affect the quality of life of these patients adversely. Oral premalignant lesion patients have a high risk of developing OC. Therefore, identifying robust survival subgroups among them may significantly improve patient therapy and care. This study aimed to identify prognostic biomarkers that predict the time-to-development of OC and survival stratification for patients using state-of-the-art machine learning and deep learning.

**Methods:**

Gene expression profiles (29,096 probes) related to 86 patients from the GSE26549 dataset from the GEO repository were used. An autoencoder deep learning neural network model was used to extract features. We also used a univariate Cox regression model to select significant features obtained from the deep learning method (*P* < 0.05). High-risk and low-risk groups were then identified using a hierarchical clustering technique based on 100 encoded features (the number of units of the encoding layer, i.e., bottleneck of the network) from autoencoder and selected by Cox proportional hazards model and a supervised random forest (RF) classifier was used to identify gene profiles related to subtypes of OC from the original 29,096 probes.

**Results:**

Among 100 encoded features extracted by autoencoder, seventy features were significantly related to time-to-OC-development, based on the univariate Cox model, which was used as the inputs for the clustering of patients. Two survival risk groups were identified (*P* value of log-rank test = 0.003) and were used as the labels for supervised classification. The overall accuracy of the RF classifier was 0.916 over the test set, yielded 21 top genes (FUT8-DDR2-ATM-CD247-ETS1-ZEB2-COL5A2-GMAP7-CDH1-COL11A2-COL3A1-AHR-COL2A1-CHORDC1-PTP4A3-COL1A2-CCR2-PDGFRB-COL1A1-FERMT2-PIK3CB) associated with time to developing OC, selected among the original 29,096 probes.

**Conclusions:**

Using deep learning, our study identified prominent transcriptional biomarkers in determining high-risk patients for developing oral cancer, which may be prognostic as significant targets for OC therapy. The identified genes may serve as potential targets for oral cancer chemoprevention. Additional validation of these biomarkers in experimental prospective and retrospective studies will launch them in OC clinics.

## Introduction

Oral cancer is among the 10th most prevalent cancer types among men and the 12th most prevalent cancer among women worldwide [1]. Oral cancer is mainly observed in the tongue, nevertheless, it can occur on many sites including the gingiva, palate, lips as well as the floor of the mouth, cheeks, and the area behind the wisdom teeth [2]. Oral squamous cell carcinoma (OSCC) accounts for over 90% of head and neck cancers (with ~ 450,000 new cases annually) [3, 4]. The incidence and deaths due to oral cancer deaths vary across the world, with the highest in developing countries [5]. Studies have shown that regardless of the advancements in screening approaches and pharmacological treatments, the incidence and mortality rates of oral cancer are practically increasing [6, 7]. Detecting oral cancer in its early stages, as well as timely treatment of the disease, are considered the most efficient ways of controlling the mortality rate [1]; nevertheless, most the oral tumors are diagnosed at an advanced stage which reduces the patients’ survival [8]. There are various treatments for OSCC, including surgery, radiotherapy, and adjunct chemotherapy (sometimes in combination), depending on the stage of the disease. However, OSCC has a poor prognosis, so the five-year overall survival rate of OSCC is less than 50% (ranges from 15 to 60%) [9, 10], depending on the severity of the disease [11].

The role of several factors including smoking, age, alcohol consumption, infections sustained by human papillomaviruses (HPVs), Epstein-Barr virus (EBV), or Candida albicans in developing OSCC, has been well-established [12–14]. Several studies have proposed some biomarkers for diagnosing oral cancer lesions with somehow low sensitivity/specificity for effective diagnosing of all oral tumors [15]. However, only 15% of all pharmaceutical agents have demonstrated “sufficient safety and potency to gain any sort of regulatory consent” [16]. Moreover, the biomarkers associated with the time-to-OSCC development (patient survival as the objective) that can be used in the prognosis of OSCC have not been fully understood and remained obscure. This indicates the deficiencies in the understanding of cancer complexity and highlights the importance of the identification of new prognostic biomarkers to obtain information for monitoring patients effectively and managing the treatment process [16]. Therefore, discovering biomarkers based on gene profiles data that are involved in the development of OSCC and improvement of survival prediction using state-of-the-art models is much needed in patients with oral preneoplastic lesions. This provides models that can mimic “the diversity of human tumor biology in a preclinical arrangement” efficiently [16], which would help an improved prognosis of OSCC and early treatment.

During the past decades, a vast majority of the literature has considered expert models in ultra-high dimensional feature spaces extensively. Among them, deep learning (DL), which is an advanced computer-aided technique, has gained much attention in the medical field [17], and it has been shown to have a vital role in detecting and diagnosing different types of cancer as well as prognosis of a disease. Autoencoders are types of DL usually used for unsupervised objectives, and recently, they have received much attention for unsupervised feature extraction in survival analysis. Studies have established autoencoder as an efficient approach to produce features related to some clinical outcomes like time-to-event response [18, 19] and have utilized autoencoders for analyzing genomics and expression data in other cancers for unsupervised feature selection as inputs of survival analysis [20, 21].

DL has been widely used in various cancers to predict the survival of patients. For example, Zhang et al. conducted a study based on the features obtained by an autoencoder algorithm to identify prognostic subtypes of high-risk neuroblastoma using multi-omics data. They showed that the autoencoder outperformed other methods like the principal component method in terms of prognosis [22]. Takahashi et al. have used an autoencoder to predict the survival of patients with lung cancer using omics data. They identified survival-associated subtypes in non-small cell lung cancer (longer and shorter-surviving groups) [23]. Chaudhary et al. utilized an autoencoder in analyzing liver cancer data and demonstrated that the DL used provides robust prognostic subtypes in liver cancer using omics data. Moreover, some attempts have been made to apply DL in the diagnosis and prognosis of OSCC. Parallel to our study, Li et al. have used an autoencoder to identify molecular subtypes of OSCC focusing on immunosuppression genes. However, they have used a different pipeline. Also, Shams and Htike have used deep neural networks using feed-forward with backpropagation design to diagnose, and predict oral cancer versus healthy controls based on gene expression profiling [24]. However, the former limited their analysis to the immunosuppressive genes and the latter handled a classification problem.

Yet, not all aspects of the time-to-development of OSCC have been fully understood. Therefore, this study used an unsupervised autoencoder framework to build a model for predicting the prognosis of OSCC patients to provide a prognostic stratification for the survival of the patients and to identify potential effective biomarkers related to the prognosis of oral cancer in patients with the oral preneoplastic lesions.

## Methods

### Data source and preprocessing

GSE26549 dataset from the GEO repository related to oral cancer tissue transcriptome (generated using the Affymetrix transcript version (microarray) with platform ID GPL6244) was used. This dataset consisted of preprocessed expression data of 86 oral preneoplastic lesion (OPL) patients, and thirty-five out of the 86 patients developed oral cancer [25]. Survival time was defined as the time to develop the oral preneoplastic lesions to oral cancer in patients. The individuals with oral preneoplastic lesions not develop oral cancer were considered as censor. The quantile normalization (QN) procedure was utilized using “bestNormalize” R package.

### Feature extraction using DL framework

Here, we used the DL computational framework on gene expression profiles related to developing OSCC in patients with the oral preneoplastic lesions. An autoencoder framework was selected as the implementation of DL for feature extraction. The philosophy of autoencoder is similar to the principal component analysis, where linear combinations of the original variables are constructed. Autoencoders receive the gene expression profiles as the inputs and reconstruct the original input by combining some nonlinear functions. These combinations are then used as new features and can be used as inputs for further analysis instead of the original variables [19]. The preprocessed gene expression profiles related to 86 samples were used as the input for the autoencoder framework. An autoencoder is a feed-forward, nonrecurrent neural network that learns through unsupervised learning [26], and is trained to reconstruct the original input to its output. Let us consider $x=\left({x}_{1}.\dots .{x}_{n}\right)$ as the input vector of dimension *n* of the input layer of an autoencoder. So, the autoencoder aims to reconstruct the *x* vector by an x' vector (dimension *n*). This is done by providing successive transformations of *x* in several hidden layers. In this study, for the *i*th layer, the rectified linear activation function or ReLU activation function was used [27] between input layer x and output layer *y*, i.e.,

$$y={f}_{i}(x)=\mathrm{ReLU}\left({W}_{i}x+{b}_{i}\right)$$

where x is a vector of size *d* and *y* is a vector of size *p*. Also, ${W}_{i}$ stands for a $p\times d$ weight matrix and ${b}_{i}$ stands for the intercept vector with size p. For an autoencoder with *k* layers, x' is then given by:

$${x}^{\mathrm{^{\prime}}}={F}_{1\to k}(x)={f}_{1}^{\circ }{\dots }^{\circ }{f}_{k-1}^{\circ }{f}_{k}(x)$$

where ${f}_{k-1}{ }^{\circ }{f}_{k}(x)={f}_{k-1}\left({f}_{k}(x)\right)$ is the composed function of ${f}_{k-1}$ with ${f}_{k}$.

An autoencoder is trained so that different weight vectors of ${W}_{i}$ are obtained to optimize (minimization problem) a specific objective function like mean squared error (MSE), measuring the error between the input $x$ and the output ${x}^{\mathrm{^{\prime}}}$ as follows:

$$MSE(x\cdot {x}^{\mathrm{^{\prime}}})=\frac{1}{N}\sum_{i=1}^{N}{\left({x}_{i}-{{x}^{\mathrm{^{\prime}}}}_{i}\right)}^{2}$$

Also, an $L1$ penalty (say, ${\alpha }_{w}$) on the weight vector of ${W}_{i}$ and an $L2$ penalty (say, ${\alpha }_{a}$) on the activities of the nodes, ${F}_{1\to k}\left(x\right)$ was added to the objective function to control overfitting as follows [28]:

$$L\left(x\cdot { x}^{{{\prime}}}\right)=MSE(x\cdot {x}^{{{\prime}}})+\sum_{i=1}^{N} \left({\alpha }_{w}{\parallel{W}_{i}\parallel }_{1}+{\alpha }_{a}{\parallel {F}_{1\to i}\left(x\right)\parallel}_{2}^{2}\right)$$

In this study, the Python Keras package (https://github.com/fchollet/keras) was utilized to build an autoencoder consisting of three hidden layers (500, 100 and 500 nodes, respectively). The bottleneck layer of the autoencoder was used to extract new features from the gene profiles of oral cancer patients. Finally, the gradient descent approach [29] with 140 epochs (iterations) and 50% dropout were utilized for training the autoencoder as the learning algorithm. Each instance of training data is processed once by the learning algorithm during one epoch. Both regularization parameters of L1 and L2 were obtained 0.0001 through cross-validation. A number of 140 epochs were used.

### Hierarchical clustering and feature selection

The extracted features from the autoencoder were examined through the univariate Cox proportional hazards (Cox-PH) model [30] to select significant features ($P<0.05$). Then, the significant features were used to cluster the patients through the hierarchical clustering algorithm [31].

### Identifying low and high-risk groups

The Kaplan–Meier curve and the log-rank test were used to identify survival groups (high-risk and low-risk groups). According to Kaplan Meier's curve, the patients with lower median survival were considered as the high-risk survival patients, and the other group was regarded as the low-risk group [32]. Also, the prognostic index ($\beta^{\prime}X$) was calculated for the external validation set, where $\beta^{\prime}$ stands for the regression coefficients obtained from a multivariate Cox regression model and X indicates the matrix of selected genes.

### Supervised random forest classifier

The survival groups identified in the previous step were considered new labels for the patients. A supervised random forest classifier was created to choose the risk-related genes. RF, introduced by [33], constructs many classification/regression trees through randomly selected training datasets and random subsets of predictors for predicting outcomes. The final prediction of the outcome is calculated by aggregating the predictions provided by each tree. So, higher accuracy is achieved by RF compared to a single decision tree model [34]. Also, RF provides variable importance criterion for variable selection. In this study, variable importance was used to select key genes [35]. The tenfold cross-validation technique was used to tune the parameters of the RF. Finally, the cut-off point of 0.002 was used as a criterion for gene selection.

### Gene Ontology (GO) and KEGG pathway enrichment analysis

The Database for Annotation Visualization and Integrated Discovery (DAVID) program was used for GO and KEGG pathway [36–38] enrichment analysis for 21 selected genes by the RF method to see if they have a role in biological process, cellular component, etc. The Benjamini adjusted P-value less than 0.05 was considered statistically significant. Gene ontology provides information that helps to computationally analyze and achieve knowledge about gene functions determined by large-scale molecular biology approaches and genetic experiments [2]. Pathways associated with genes are also provided in gene ontology.

### Protein–protein interaction (PPI) network analysis

The PPI network was constructed using the “Search Tool for the Retrieval of Interacting Genes” (STRING) for 21 selected genes. A confidence score of 0.4 was considered as a threshold for network construction. Afterward, the PPI network was visualized and analyzed by Cytoscape software (V 3.8.2).

## Results

Thirty-five out of 86 patients developed oral cancer. The mean and median follow-up time of the patients were 9.01 and 10.7 years (min = 0.18 and max = 14.34 years), respectively. One-, three- and five-year survival rates of the patients were 88%, 73%, and 65%, respectively.

**Fig. 1:**
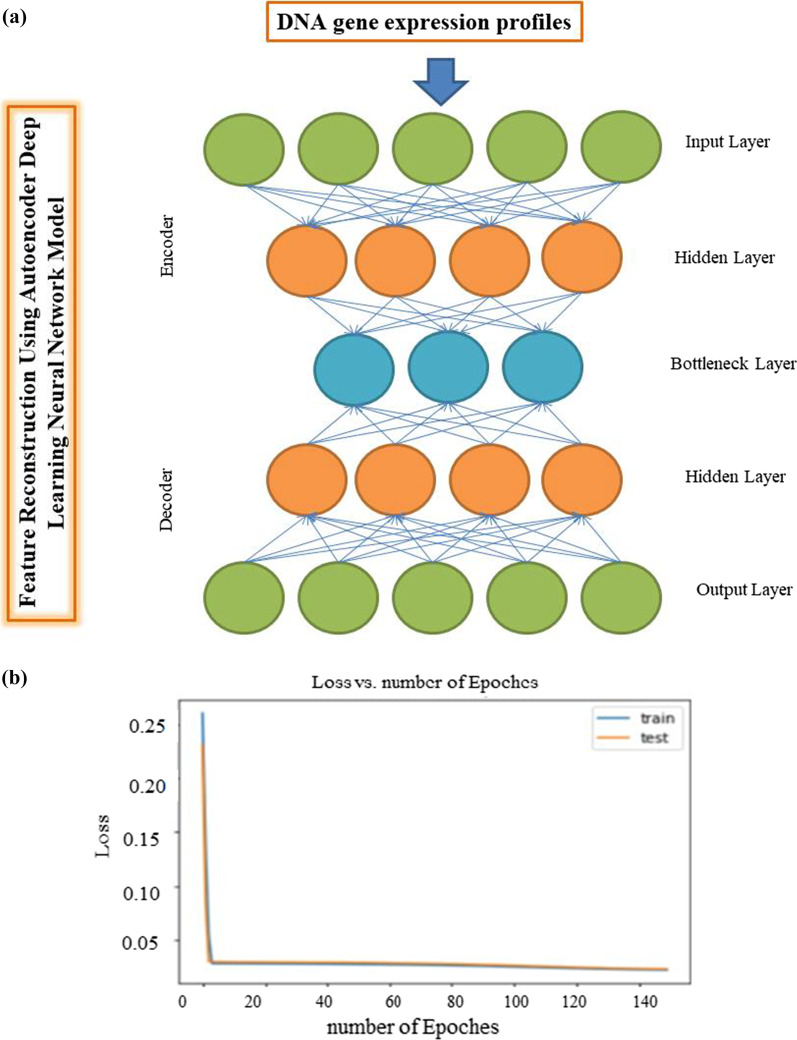
**a** Architecture of the autoencoder, and **b** loss function values over epochs

**Fig. 2:**
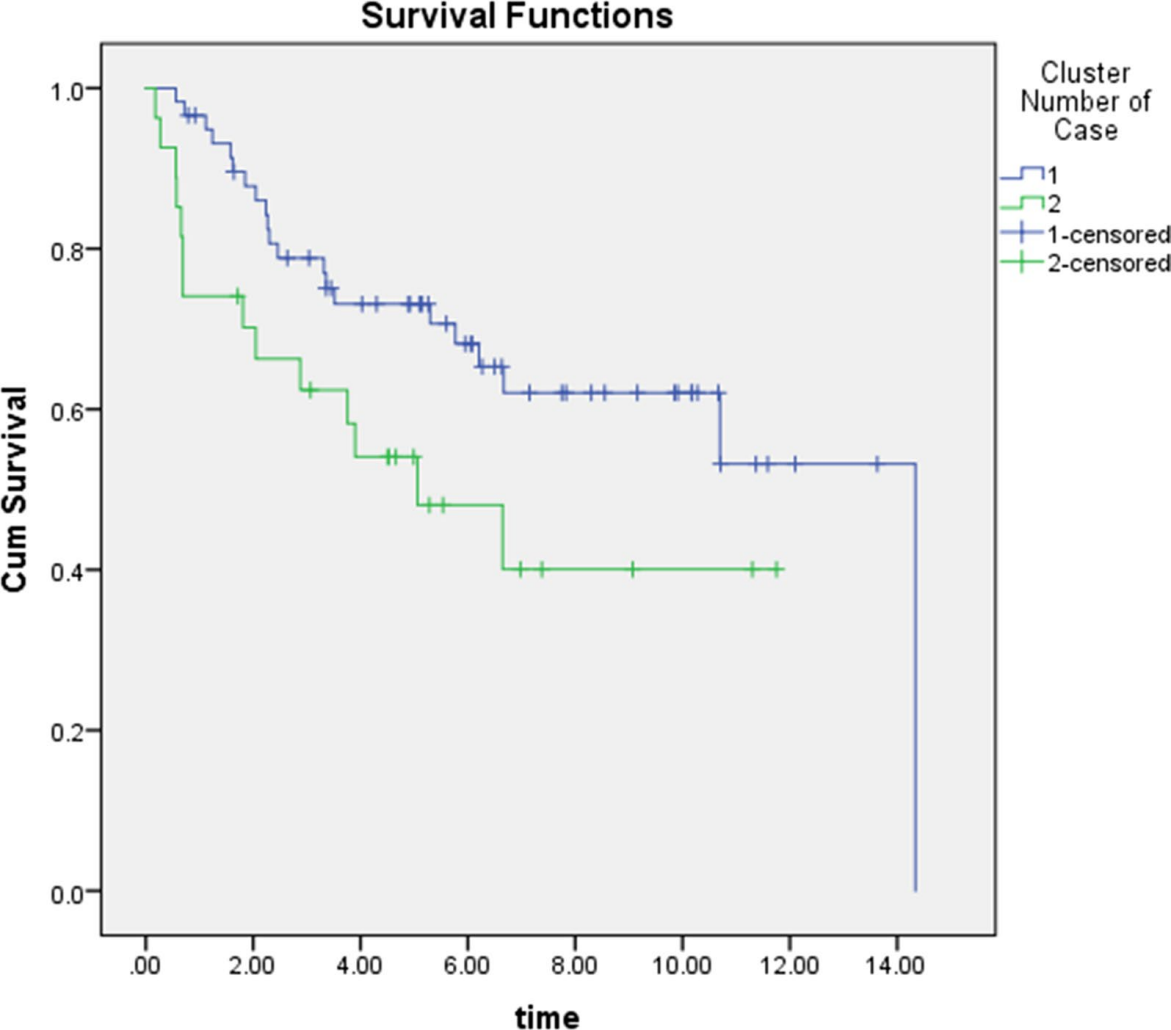
Kaplan Meier curve for two subgroup of survival time

The information on gene expression profiles related to 86 patients with oral preneoplastic lesions was used as input features of the autoencoder, a DL framework. Figure 1 illustrates the architecture of the autoencoder (a) and the loss values (MSE) versus the epochs (b). The activity of the 100 nodes from the bottleneck hidden layer was extracted as new features. Seventy features out of the 100 new features were statistically significant using univariate Cox-PH regression (*P* < 0.05), and they were shown to be associated with the survival of the patients. These 70 features were subjected to hierarchical clustering, with cluster number K ranging from 2 to 6. Considering the silhouette index, the number of 2 (k = 2) clusters was the optimum. Table 1 shows the characteristics of each group identified using clustering based on 70 features from DL. The median survival for group 1 was ~ 5 years and ~ 10 years. Furthermore the survival analysis on the entire data (86 patients) showed that the survival curves in the two identified clusters (Fig. 2) were statistically different (log-rank test *P* = 0.003). We also conducted the penalized principal component analysis as an alternative to the DL. Using univariate Cox regression, 20 out of 100 principal components were significant (*P* < 0.05). The same strategy was used to identify two groups, but the difference between the two survival curves using this method was not statistically significant (*P* = 0.171). Thus, the two classes were considered labels for the subsequent supervised RF classifier.

**Table 1:** Survival information of two identified groups

Subgroup	NO. Patients (%)	NO. events (%)	NO. censor (%)	Mean (Year)	SE	Median (Year)
1	27 (31.4)	16 (59.3)	11 (40.7)	5.33	0.90	5.06
2	59 (68.6)	19 (32.2)	40 (67.8)	10.23	0.81	10.76

**Fig. 3:**
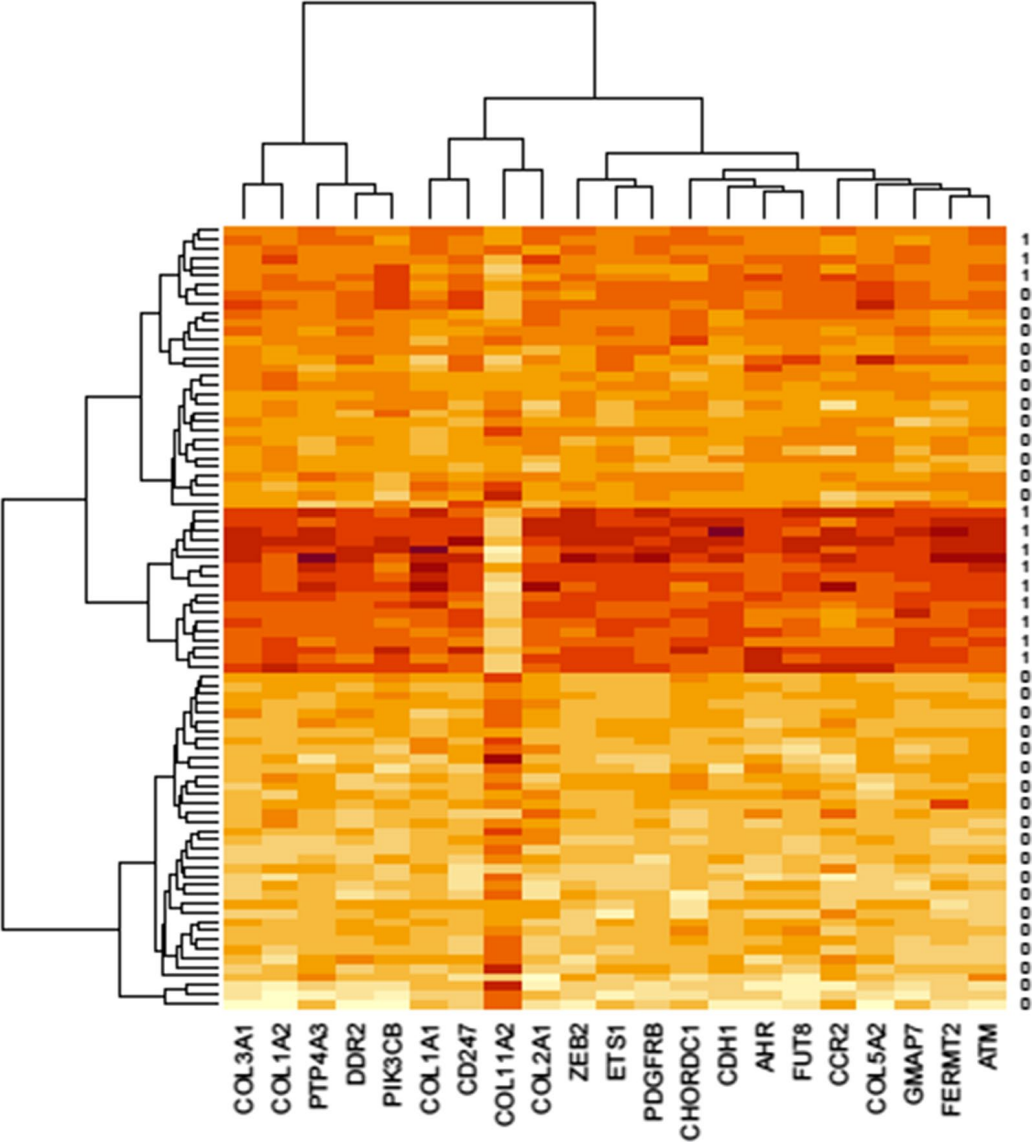
Heat-map of the 21 selected genes using random forest related two identified survival groups

The RF classifier was trained (sensitivity = 0.814, specificity = 0.966, and total accuracy = 0.916 over the 30% of data as a testing set), and gene profiles related to the survival risk groups were selected based on variable importance greater than 0.002. Table 2 shows the variable importance of 21 top-rank genes with variable importance greater than 0.002 and their over/under expression status in high-risk patients. Also, Fig. 3 illustrates the heatmap of the 21 selected genes.

**Table 2:** Top genes identified by random forest method through variable importance (VIMP)

Order	Probe set	VIMP	Gene symbol	Value in high-risk group
1	8103389	100	FUT8	Overexpressed
2	8137240	75	DDR2	Overexpressed
3	8128956	46	ATM	Overexpressed
4	8055639	36	CD247	Overexpressed
5	8175393	35	ETS1	Overexpressed
6	7903358	32	ZEB2	Overexpressed
7	8055624	31	COL5A2	Overexpressed
8	8101260	27	GMAP7	Overexpressed
9	7926127	26	CDH1	Overexpressed
10	8002218	21	COL11A2	Underexpressed
11	8137244	21	COL3A1	Overexpressed
12	8003667	18	AHR	Overexpressed
13	8098637	16	COL2A1	Overexpressed
14	8138805	14	CHORDC1	Overexpressed
15	7943620	12	PTP4A3	Overexpressed
16	7953603	11	COL1A2	Overexpressed
17	7957277	10	CCR2	Overexpressed
18	8045563	9	PDGFRB	Overexpressed
19	7953835	7	COL1A1	Overexpressed
20	8171684	7	FERMT2	Overexpressed
21	7929511	6	PIK3CB	Overexpressed

### Gene Ontology (GO) and KEGG pathway enrichment analysis

**Fig. 4:**
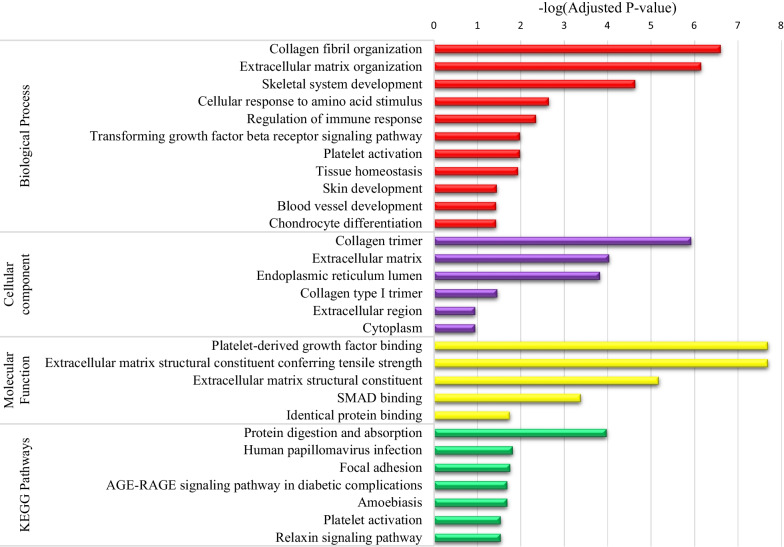
Summary of the top GO results and KEGG pathways

Summary of the top GO results and KEGG pathways were illustrated in Fig. 4. The results of GO enrichment analysis indicated that collagen fibril organization, extracellular matrix organization, skeletal system development, cellular response to amino acid stimulus, regulation of immune response, transforming growth factor beta receptor signaling pathway, platelet activation, tissue homeostasis, skin development, blood vessel development, and chondrocyte differentiation terms were significantly enriched in biological process (BP). Collagen trimer, extracellular matrix, endoplasmic reticulum lumen, collagen type I trimer, extracellular region, and Golgi apparatus were significantly enriched in cellular component (CC). Platelet-derived growth factor binding, extracellular matrix structural constituent conferring tensile strength, extracellular matrix structural constituent, SMAD binding, and identical protein binding terms were significantly enriched in molecular function (MF).

The KEGG pathway analysis indicated that the protein digestion and absorption, human papillomavirus infection, focal adhesion, AGE-RAGE signaling pathway in diabetic complications, amoebiasis, platelet activation, and relaxin signaling pathway terms were enriched for 21 selected genes (Fig. 4).

### PPI network analysis

**Fig. 5:**
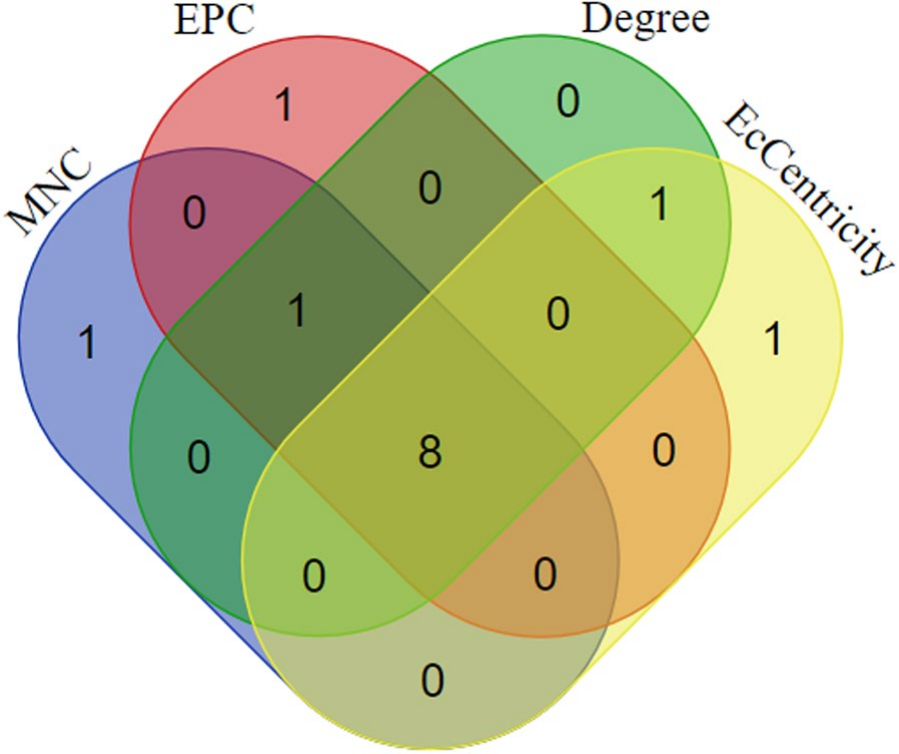
The overlap between the top predicted target genes, ranked by MNC, MCC, and degree, is illustrated in a Venn diagram

**Fig. 6:**
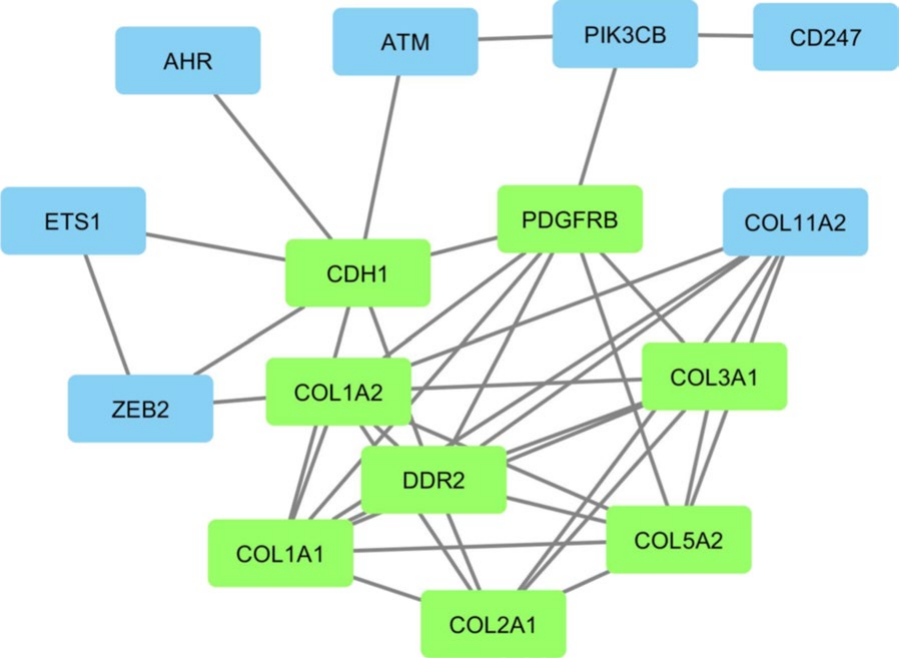
The PPI network of identified genes, formed by using Cytoscape software. Proteins are represented by nodes, and interactions between two proteins are described by edges

The constructed PPI network was composed of 15 nodes and 38 edges. In order to find the hub genes, including in the pathogenesis of oral cancer, the constructed PPI network was evaluated with the CytoHubba package under Cytoscape software. The top 10 genes were identified using the four methods, including degree, MNC, EPC, and EcCentricity. After depicting the Venn diagram (Fig. 5), eight common genes including *PDGFRB, COL1A2, CDH1, DDR2, COL3A1, COL2A1, COL1A1,* and *COL5A2* were selected as hub genes (Fig. 6).

### In silico validation of selected genes

**Fig. 7:**
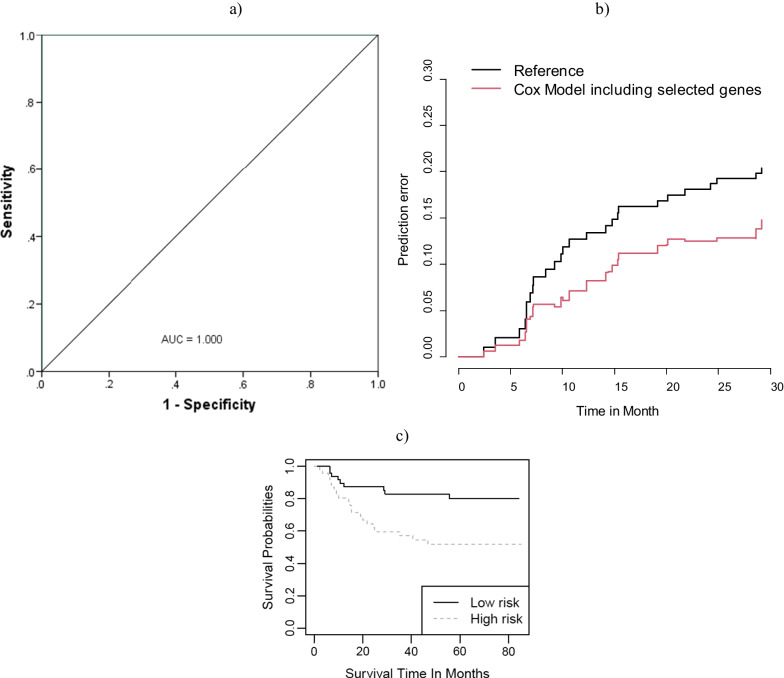
**a** ROC curve related to the prediction of oral cancer patients and healthy controls in in silico validation data set (GSE9844); **b** prediction error curve in predicting survival of oral cancer patients over GSE41613 data set as in silico validation; **c** Kaplan-Mayer curves of survival subgroups identified using selected genes over GSE41613 data set as in silico validation

An in silico validation was conducted for the selected probes using two public data sets with series accession numbers of GSE9844 and GSE41613 on oral cancer, so that we predicted the outcome of independent samples related to external data sets based on the selected genes from the previous steps using the utilized method in the original data set (GSE26549). The first dataset included gene expression profiles of 26 microdissected OTSCC tissues and 12 matching normal tissue samples [39]. We applied the RF method for the classification of the oral cancer patients and healthy controls. About 70% of the data was considered as a training set, and the rest of them were used to test the method. On the training data set, a three-fold cross-validation strategy was used to tune the parameters. Figure 7a depicts the ROC curve along with the AUC for the testing set in the in silico validation data set using the selected genes in Table 2. According to the results, the AUC was 1.000, indicating that the identified genes can successfully predict oral cancer development and can be used for the prognosis of the patients.

The second data set included the survival time of 97 oral cancer patients. The prediction error curve, based on a model including selected genes in the previous steps, was provided in Fig. 7b indicating that the selected genes were potentially informative in predicting the survival of the patients with oral cancer. Also, we calculated the prognostic index ($\beta^{\prime}X$, where $\beta^{\prime}$ stands for the regression coefficients obtained from a multivariate Cox regression model, and X indicates the matrix of selected genes). So, the patients were divided into two risk groups. Figure 7c illustrates the Kaplan–Meier survival curves for the two groups. The log-rank test showed a statistical difference between the curves (Chisq = 8.1 on 1 degree of freedom, *P* = 0.004).

## Discussion

According to the findings of the present study, *FUT8* (Fucosyltransferase 8) was the first top gene identified by the algorithm used (DL and RF). FUT8 is a protein-coding gene that encodes an enzyme belonging to the family of fucosyltransferases involved in many pathological/physiological activities such as tumor metastasis and inflammation) [40, 41], and regulating the fucosylation of O-glycans and N-glycans [40]. Results of the present study indicated overexpression of FUT8 in identified high-risk patients compared to the low-risk group, which was in concordance with other studies [42]. Studies have shown increasing mRNA levels of *FUT8* and core glycoprotein in tumor tissues of oral cancer patients compared with normal oral epithelial/oesophageal tissue [42, 43]. “*FUT8* plays an anti-radiation-driven role in ESCC by core fucosylation of CD147, and it can be used as a marker to predict the radiotherapy response of *ESCC* patients” [40, 44]. According to the findings, *DDR2* (Discoidin domain receptor 2) was the second top rank gene identified by RF, showing overexpression in high-risk patients. This finding was in concordance with the results of other studies [45, 46]. *DDR2* is a receptor tyrosine kinase (RTK), and it has been shown to be activated through fibrillar collagens [46] and involved in cell behaviors of different types of cancer, including *VEGF* expression, differentiation, tumor angiogenesis, invasion, and metastatic potential of *HNSCC* cell lines [46]. *DDR2* has been well-established to be activated through binding with collagens. Then, a series of intracellular pathways of *p38, JNK, ERK1/2, Notch-1,* and *NF-κB* are activated [47, 48]. Several studies have shown the regulatory functions of *DDR2* factor in different types of cancers, including lung carcinoma [49]. Ataxia telangiectasia mutated (ATM) was the third top rank gene identified as an important gene in determining high-risk patients. It was shown that it is over-expressed in high-risk survival group patients. This finding was consistent with the results of other studies [50]. *ATM* encodes a vital cell cycle checkpoint (CCK) kinase protein belonging to the PI3/PI4-kinase family that functions as a regulator of various downstream proteins, including “tumor suppressor proteins p53 and *BRCA1*, checkpoint kinase *CHK2*, checkpoint proteins *RAD17* and *RAD9*, and DNA repair protein *NBS1*”. This protein is thought to be one of the two master controllers of *CCK* signaling pathways essential in cell response to DNA damage and genome stability [51].

The protein encoded by *PDGFRB* as a plasma membrane receptor belongs to the platelet-derived growth factor family. The binding of *PDGF* ligands to this receptor leads to dimerization and activation of downstream signaling pathways having a role in the regulation of motility and proliferation, differentiation, and survival of cells [52]. Lin et al., in their study, indicated that *PDGFRB* expression level was associated with poor prognosis and lymph node metastasis of OSCC [53]. E-cadherin protein encoded with *CDH1* belongs to the cadherin protein family. This transmembrane glycoprotein, which regulates cell adhesion, is a tumor suppressor protein [54]. Pannone et al., in their study, showed that the expression level of *CDH1* decreases in oral tumors in mRNA and protein levels. Moreover, the expression level of E-cadherin had a reverse correlation with tumor grade and prognosis of patients [55].

The protein encoded with *COL1A2* and *COL1A1* is the building block of type I collagen. The expression level of *COL1A2* is dysregulated in several tumors such as malignant melanoma head and neck ovarian pancreatic, and bladder cancer [56, 57]. *COL3A1* encoded the polypeptide chain which was the building block of type III collagen. The expression of this gene has an essential role in the proliferation and migration of tumor cells and is dysregulated in several malignancies, such as ovarian cancer and brain tumor [58, 59]. Collagen, type II, alpha 1 peptide encoded with *COL2A1* form the homotrimer Type II collagen [60]. Tarpey et al., in a study, showed that in Chondrosarcoma, hyper-mutability of *COL2A1* is common [61]. Moreover, Ganapathi et al., in a study, indicated that the expression level of this gene is associated with the prognosis of high-grade serous ovarian cancer [62]. Type V collagen consists of peptides encoded with *COL5A2*. This gene has an essential role in the regulation of angiogenesis and metastasis of several tumors such as osteosarcoma, colorectal cancer, gastric cancer, and breast cancer [63, 64].

The results of GO enrichment analysis indicated that genes identified in this study representing biological pathways were significantly enriched in relation to cancers. These findings were in agreement with similar studies, so that cancer-related terms such as collagen fibril organization, extracellular matrix organization [65], cellular response to amino acid stimulus, platelet activation [66], tissue homeostasis [67], regulation of immune response, skin development [68], platelet-derived growth factor binding, extracellular matrix structural constituent, SMAD binding [66], extracellular matrix [65], collagen trimmer, and endoplasmic reticulum lumen [66] were enriched for 21 selected genes by the RF method. Moreover KEGG pathway enrichment analysis indicated that cancer related pathways such as protein digestion and absorption, platelet activation [65], focal adhesion, Amoebiasis [66], human papillomavirus infection [69], AGE-RAGE signaling pathway in diabetic complications [70], and relaxin signaling pathway [71] were significantly enriched which was in agreement with the results of other studies.

In the present study, we sued a univariate Cox regression model as the multivariate regression could not be applied here due to a large number of unsupervised extracted features (> 100) compared to the sample size (n = 86). It is suggested to use other methods of screening selected features, like penalized Cox regression model with different penalties to choose a subset of features among the pool of features, and to conduct simulation studies to see which variable selection method works better.

## Conclusion

This study identified eight hub genes, including *PDGFRB, COL1A2, CDH1, DDR2, COL3A1, COL2A1, COL1A1,* and *COL5A2,* that may have a role in development of oral cancer. Further experimental investigations are required in order to well-understand and to validate the pathogenic role of these genes in oral cancer.

## Data Availability

The datasets is publically available on https://www.ncbi.nlm.nih.gov/geo/. All analyzed during the current study are available from the corresponding author on request.
